# Relationship between expression of PD-L1 and tumor angiogenesis, proliferation, and invasion in glioma

**DOI:** 10.18632/oncotarget.17922

**Published:** 2017-05-17

**Authors:** Song Xue, Man Hu, Peifeng Li, Ji Ma, Li Xie, Feifei Teng, Yufang Zhu, Bingjie Fan, Dianbin Mu, Jinming Yu

**Affiliations:** ^1^ School of Medicine and Life Sciences, University of Jinan-Shandong Academy of Medical Sciences, Jinan, Shandong, China; ^2^ Shandong Academy of Medical Sciences, Jinan, Shandong, China; ^3^ Department of Radiation Oncology, Shandong Province Key Laboratory of Radiation Oncology, Shandong Cancer Hospital and Institute, Jinan, Shandong, China; ^4^ Shandong Cancer Hospital Affiliated to Shandong University, Jinan, Shandong, China; ^5^ Department of Pathology, General Hospital of Jinan Military Command, Jinan, Shandong, China; ^6^ Department of Medicine, Shandong Cancer Hospital and Institute, Jinan, Shandong, China; ^7^ Shandong Provincial Key Laboratory of Radiation Oncology, Shandong Cancer Hospital and Institute, Jinan, Shandong, China; ^8^ Department of Neurosurgery, Shandong Cancer Hospital and Institute, Jinan, Shandong, China; ^9^ Department of Pathology, Shandong Cancer Hospital and Institute, Jinan, Shandong, China

**Keywords:** PD-L1, angiogenesis, proliferation, invasion, glioma

## Abstract

Programmed death ligand 1 (PD-L1) is highly expressed in many cancers. We investigated the expression of PD-L1 and its relationship with vascular endothelial growth factor (VEGF), matrix metalloproteinase-9 and KI-67 expression in 64 patients with primary glioma. The expression rate of PD-L1 in glioma patients was 78.12%. PD-L1 levels correlated with the tumor grade (*p* = 0.013), VEGF status (*p* = 0.002) and KI-67 status (*p* = 0.002). In addition, PD-L1 levels correlated positively with VEGF (*r* = 0.314, *p* = 0.011) and KI-67 (*r* = 0.391, *p* = 0.001) levels when the data were treated as continuous variables. This is the first report suggesting that PD-L1 is important for glioma angiogenesis and proliferation. Thus, further research should be conducted to assess the combination of targeted VEGF therapy and anti-PD-L1 immunotherapy for the treatment of glioma.

## INTRODUCTION

Glioma is the most common type of primary brain tumor of the central nervous system, accounting for 40–50% of all primary brain tumors. Despite advances in therapeutic approaches such as surgery, radiotherapy and chemotherapy over the past decade, the overall survival of glioma patients remains dismal [1, 2]. In particular, patients with glioblastoma (GBM) survive for a median of 14.6 months after diagnosis, and their five-year survival rate is less than 5% [3]. It is well known that gliomas can suppress immune responses by downregulating antigen presentation [4], upregulating anti-inflammatory proteins [5], and promoting the expansion of immunosuppressive effector cells [6]. Accordingly, glioma can create an immunosuppressive environment and evade immune surveillance by multimodal mechanisms.

Programmed death ligand 1 (PD-L1), which binds to programmed death 1 (PD-1) on T cells, B cells, dendritic cells and natural killer T cells, provides tumor cells an important escape mechanism from immune attack, thus leading to immune suppression [7, 8]. In the past five years, anti-PD-1 or anti-PD-L1 antibodies blocking the binding between PD-1 and PD-L1 have been reported to promote marked antitumor immunity, and have risen to the forefront of immunotherapy due to their notable clinical efficacy in melanoma and non-small cell lung cancer clinical trials [9, 10]. Although treatments targeting PD-1/PD-L1 have had enormous success in cancer therapy, and immunotherapies exhibit more durable clinical activity than conventional chemotherapy, the objective response rate to these treatments has only been 6–14.5% in patients with advanced cancers such as ovarian cancer, renal-cell cancer and squamous non-small cell lung cancer [11, 12].

Aberrant PD-L1 expression has been reported to occur in glioma and to contribute to immunoresistance [8]. In preclinical studies, antibodies targeting PD-1/PD-L1 have been successful in animal models of glioma [13]. Phase II and III clinical trials (NCT02017717 and NCT01952769) are ongoing for these promising immunotherapies in glioma patients, including those with GBM.

PD-L1 expression tightly correlates with the pathological grade of glioma [14, 15]; thus, proteins involved in angiogenesis, proliferation and invasion, which are associated with the malignant progression of glioma, might also regulate PD-L1 expression. Angiogenesis, a dynamic process required to sustain tumor cell growth and metastasis, is mainly induced by vascular endothelial growth factor (VEGF). VEGF expression has been found to be upregulated in glioma and to correlate with tumor malignancy [16, 17]. In addition to promoting angiogenesis and vascular permeability, increased VEGF expression may also contribute to an immunosuppressive tumor microenvironment [18]. For example, PD-L1 expression was upregulated in myeloid dendritic cells incubated with VEGF secreted by primary ovarian carcinoma cell lines [19]. However, the data on the relationship between PD-L1 and VEGF have been limited and inconsistent. In clear cell renal cell carcinoma, PD-L1 expression was positively associated with VEGF expression [20]. However, Joseph *et al*. reported a significant inverse correlation between the expression of PD-L1 protein and VEGF-related genes [21]. The expression of PD-L1 and VEGF in GBM are important characteristics that remain to be clearly defined. To the best of our knowledge, the correlation between PD-L1 and VEGF expression in glioma patients has not been reported.

Matrix metallopeptidase 9 (MMP-9), belonging to the zinc-metalloproteinase family involved in the degradation of the extracellular matrix, appears to facilitate the initiation and progression of multiple biological events required for glioma progression, such as invasion and migration of glioma cells. MMP-9 was also shown to proteolytically cleave PD-L1 in fibroblasts, thus suppressing T cell apoptosis [22].

KI-67 is the most widely used marker for proliferation in clinical practice. PD-L1 expression was found to correlate strongly with KI-67 expression (i.e., high tumor cell proliferation) in breast cancer patients [23]. However, there are limited data regarding the association of PD-L1, MMP-9 and KI-67 expression in glioma.

We hypothesized that angiogenesis, invasion and proliferation might be important regulators of the PD-L1/PD-1 axis in glioma, given the association of these processes with the malignant progression of glioma. Thus, we investigated the protein profiles of PD-L1, VEGF, MMP-9 and KI-67 in glioma patients according to the patients' clinical characteristics, and explored the correlations in the expression of these proteins.

## RESULTS

### The mean optical density (MOD) of PD-L1, VEGF, MMP-9 and KI-67 in glioma

**Figure 1:**
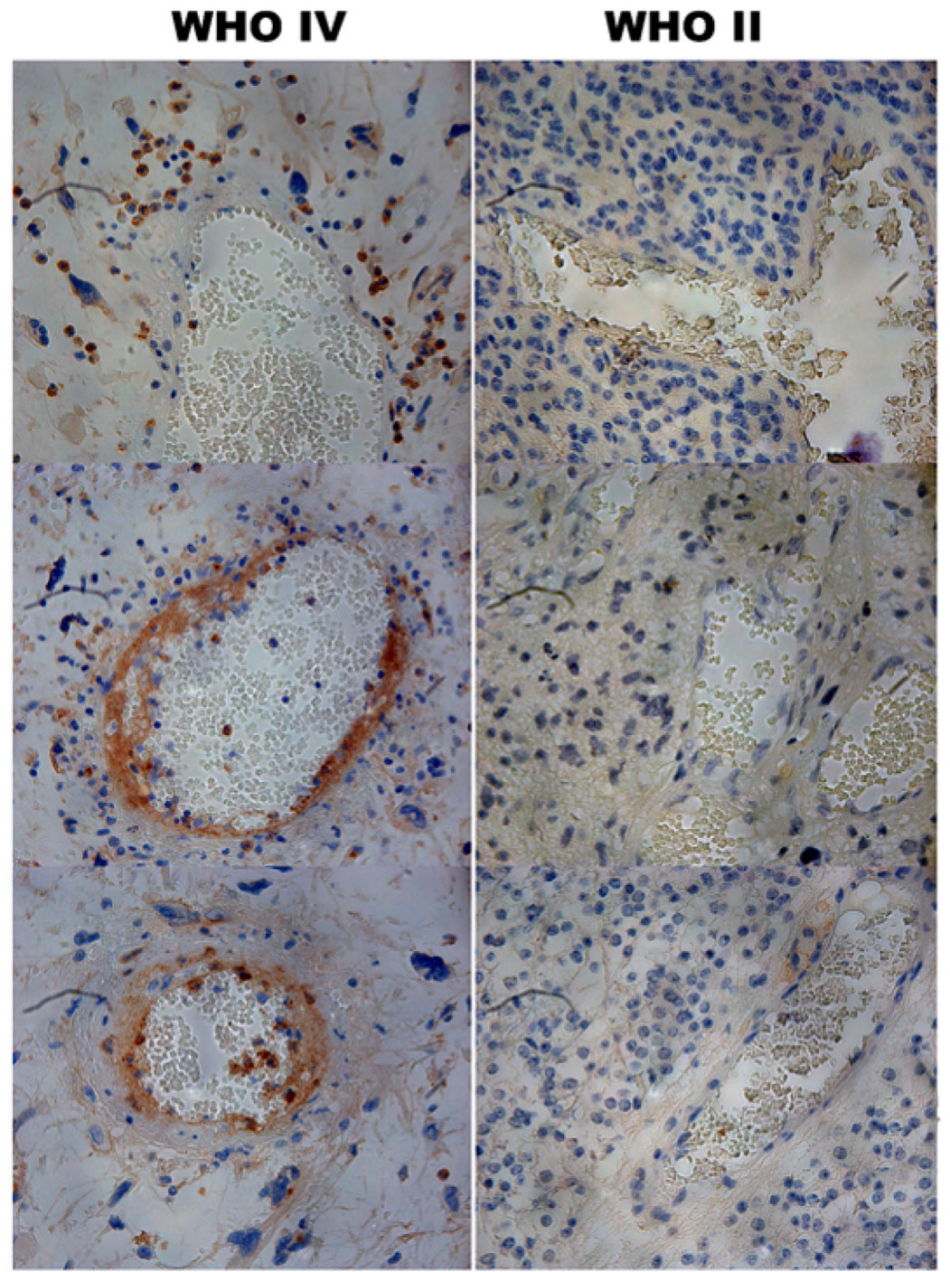
Immunohistochemical staining of PD-L1 in human glioma tissues Staining of PD-L1 was observed around or in the blood vessels of the high grade gliomas (×400).

**Figure 2:**
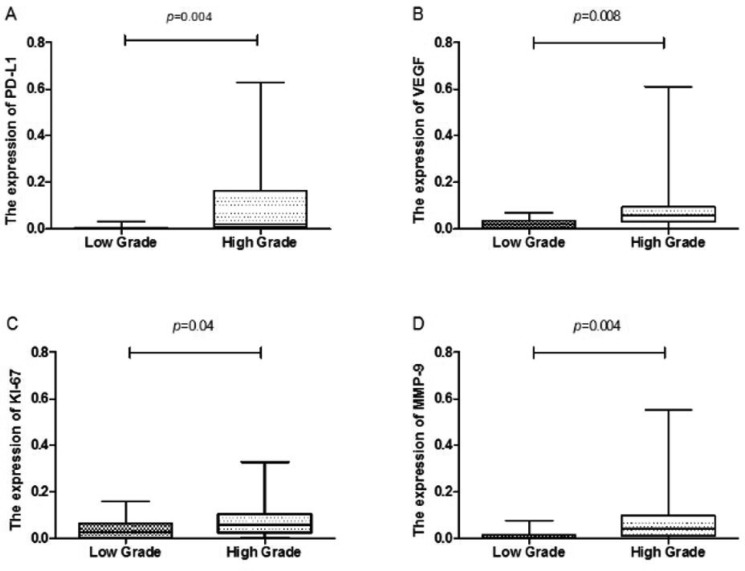
Box plot comparing PD-L1, VEFG, MMP-9 and Ki-67 activity in patients with low grade gliomas and those with high grade gliomas The horizontal lines within the boxes represent median values, and the boxes denote the interquartile range. Vertical lines denote the full ranges.

Using the 2016 World Health Organization (WHO) classification of tumors of the central nervous system, we recruited a total of 23 low-grade glioma (LGG) (WHO I–II) and 41 high-grade glioma (HGG) (WHO III - IV) patients for this study. tumor (Figure 1), indicating that PD-L1 was associated with strong invasion and migration abilities. By using Image-Pro Plus, we found that PD-L1 immunoreactivity varied considerably among the 64 patients; the MOD ranged from 0.00000311 to 0.6276, and the mean across samples was 0.07511 ± 0.01877. The mean MOD in HGG (0.1144 ± 0.02754) was greater than that in LGG (0.005129 ± 0.001441; *p* = 0.0044) (Table 1 and Figure 2).

**Table 1:** Mean (± SD) MOD expression of PD-L1, VEFG, MMP-9 and Ki-67 in glioma specimens from LGG and HGG

Index	Mean of MOD (means ± SD)		*p*^a^
	LGG (*n* = 23)	HGG (*n* = 41)	
PD-L1	0.005129 ± 0.001441	0.1144 ± 0.02754	0.0044
VEGF	0.02248 ± 0.004159	0.09598 ± 0.01981	0.0078
MMP	0.04203 ± 0.009844	0.07884 ± 0.01191	0.0401
Ki-67	0.01151 ± 0.003914	0.08850 ± 0.01914	0.0041

^a^The difference in MOD count between the two groups was assessed using a *t*-test.

Abbreviations: SD: Standard deviations; MOD: Mean optical density; PD-L1: Programmed Death Ligand 1; VEGF: Vascular endothelial growth factor; MMP-9: Matrix metallopeptidase-9, LGG: Low grade glioma; HGG: High grade glioma.

**Figure 3:**
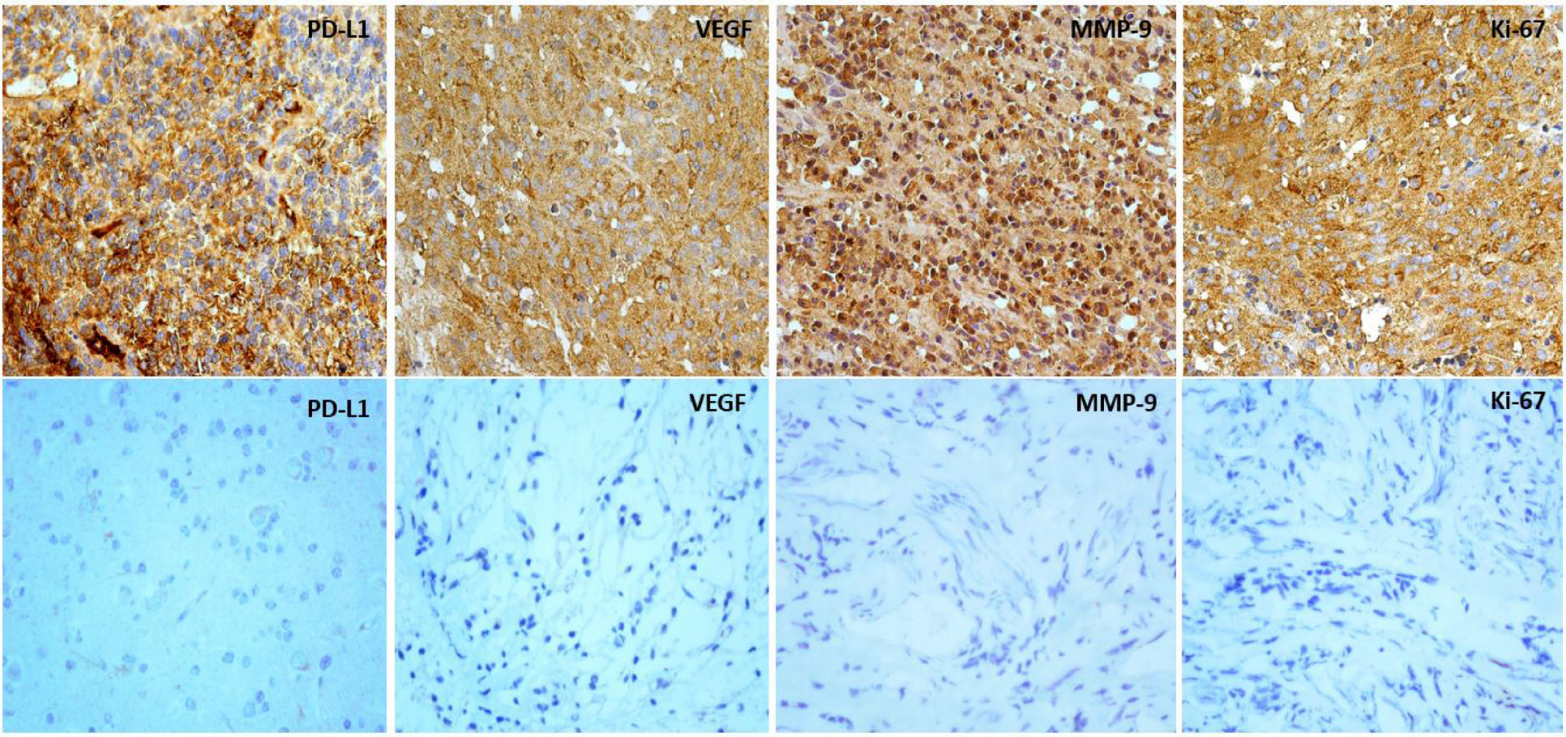
Expression of PD-L1, VEGF, MMP-9 and Ki-67 in human glioma tissues Upper: expression of PD-L1, VEGF, MMP-9 and Ki-67 in high pathological grading of gliomas illustrated by immunostained sections (×400). Lower: expression of PD-L1, VEGF, MMP-9 and Ki-67 in low pathological grading of gliomas illustrated by immunostained sections (×400).

The mean MODs for VEGF, MMP-9 and KI-67 staining were also significantly higher in HGG (0.09598 ± 0.01981, 0.07884 ± 0.01191, and 0.08850 ± 0.01914, respectively) than in LGG (0.02248 ± 0.004159, 0.04203 ± 0.009844, and 0.01151 ± 0.003914, respectively) (*p* = 0.0078, 0.0401, and 0.0041, respectively; Table 1 and Figure 2). Representative images of PD-L1, VEGF, MMP-9 and KI-67 staining in HGG tissue are shown in Figure 3.

### Relationship between PD-L1 expression and clinicopathological features

Overall, PD-L1 protein expression was observed in 78.12% (50/64) of patients with glioma. PD-L1 was expressed in 60.87% (14/23) of LGG cases and 87.80% (36/41) of HGG cases. The correlation of PD-L1 expression with clinicopathological features was assessed with a Mann–Whitney U test. PD-L1 immunopositivity was significantly associated with the pathological grade (*p* = 0.013), VEGF status (*p* = 0.002) and KI-67 index (*p* = 0.002). Positive VEGF and KI-67 expression were more significant frequently observed in the PD-L1 positive group (85.19% and 86.27%) than in the PD-L1 negative group (14.81% and 13.73%), respectively. In addition, MMP-9 expression was more frequently detected in the PD-L1 positive group (82.69%) than in the PD-L1 negative group (14.81%), although the difference was not statistically significant (*p* = 0.068). PD-L1 expression was not significantly associated with clinicopathological features such as gender, age, and Karnofsky Performance Status (*p* = 0.183, 0.580, and 0.080, respectively). The correlations between PD-L1 expression and clinicopathological features are shown in Table 2.

**Table 2:** Clinical and pathologic information

		*N*	Expression of PD-L1(%)		*p*^a^
			Negative	Positive	
Total		64	14 (21.88)	50 (78.12)	
Gender					0.183
	Male	33	5 (15.15)	28 (84.85)	
	Female	31	9 (29.03)	22 (70.97)	
Age					0.580
	< 60	45	9 (20.00)	36 (80.00)	
	≥ 60	19	5 (26.32)	14 (73.68)	
KPS					0.080
	< 80	9	4 (44.44)	5 (55.56)	
	≥ 80	55	10 (18.18)	45 (81.82)	
WHO Grade					0.013
	LGG	23	9 (39.13)	14 (60.87)	
	PA	11	5	6	
	DA	12	4	8	
	HGG	41	5 (12.20)	36 (87.80)	
	AA	13	1	12	
	GBM	28	4	24	
VEGF					0.002
	Negative	10	6 (60.00)	4 (40.00)	
	Positive	54	8 (14.81)	46 (85.19)	
MMP-9					0.068
	Negative	12	5 (41.67)	7 (58.33)	
	Positive	52	9 (17.31)	43 (82.69)	
Ki-67					0.002
	Negative	13	7 (53.85)	6 (46.15)	
	Positive	51	7 (13.73)	44 (86.27)	

^a^The correlation between PD-L1 expression and clinicopathological features was assessed by Mann–Whitney *U* test. Abbreviations: LGG, low grade glioma; PA, pilocytic astrocytoma; DA, diffuse astrocytoma; HGG, high grade glioma; AA, anaplastic astrocytoma; GBM, glioblastoma.

### The relationship of PD-L1 expression with VEGF, MMP-9 and KI-67 expression

Because VEGF and KI-67 were more significant frequently observed in the PD-L1 positive group than in the PD-L1 negative group, we decided to analyze the relationships among PD-L1, VEGF and KI-67 levels by treating the MOD as a continuous variable. PD-L1 levels were positively associated with the levels of VEGF (*r* = 0.314, *p* = 0.011) and KI-67 (*r* = 0.391, *p* = 0.001) (Table 3). As VEGF and KI-67 levels both correlated positively with PD-L1 levels, we determined the association of these molecular markers with each other. We found a strong positive correlation between VEGF and KI-67 levels (*r* = 0.4909, *p* < 0.001) (Table 3).

**Table 3:** Spearman's correlation (*r*) for continuous variables

Variable		PD-L1	VEGF	Ki-67
PD-L1	*r*	-	0.314	0.391
	*p*		0.011	0.001
VEGF	*r*	0.314	-	0.4909
	*p*	0.011		< 0.001
Ki-67	*r*	0.391	0.4909	-
	*p*	0.001	< 0.001	

Abbreviations: *r*: rank correlation.

## DISCUSSION

The co-inhibitory characteristics of PD-L1 are attributed to its binding to its receptor, PD-1, on tumor-specific T cells, which leads to the apoptosis and exhaustion of activated immune cells [24]. PD-L1 expression has been investigated in different types of tumors, including gastric carcinoma, pancreatic carcinoma and natural killer/T-cell lymphoma. [25–27]. In pancreatic carcinoma, PD-L1 expression was found to correlate with the pathological grade and tumor-node-metastasis stage [26]. In early-stage natural killer/T-cell lymphoma patients, higher levels of PD-L1 in serum and tumor tissues were associated with shorter survival times [27]. Increased PD-L1 expression has also been observed in glioma patients and correlated with glioma grade, which demonstrates that PD-L1 may be a candidate tissue biomarker for gliomas [28–30].

In a microarray study of glioma tissues (2007 WHO grade I–IV), Zeng *et al*. found that the tumor-cell PD-L1 expression rate was 51.1% in all patients with gliomas. The PD-L1 expression rates were 49.2%, 53.7% and 68.8% for grade II, III and IV samples, respectively [31]. Nevertheless, tissue microarrays may not grossly represent tumor tissues. In this study, we retrospectively analyzed full histological slides of samples from 64 glioma patients (WHO 2016 grade I–IV). Positive PD-L1 expression was found in 78.12% of these samples (14/23 grade I – II samples, and 36/41 grade III–IV samples). The rate of PD-L1 protein expression was significantly higher in HGG than in LGG (87.80% vs. 60.87%, *p* = 0.013).

The rate of PD-L1 protein positivity has varied across studies. For example, in a study with a small sample of 10 patients, PD-L1 protein expression was detected in all nine GBM specimens (WHO IV) and one mixed glioma (WHO III) specimen [32].

Recent studies found that PD-L1 was overexpressed by GBM and the positive rate of PD-L1 protein expression was ranged from 31.5% to 61.0% [33–35]. However, PD-L1 was not detected in samples from 30 human ependymoma patients (WHO 2016 grade II–III) [36]. Differences in the sample size, WHO classification, antibody type and positivity cut-off may have contributed to the discrepancies among these studies. We used Image Pro-Plus to quantify PD-L1 staining levels precisely in the tumor tissue. We found that PD-L1 immunoreactivity varied considerably among the 64 patients, and that the mean MOD was higher in HGG than in LGG (0.1144 ± 0.02754 vs. 0.005129 ± 0.001441, *p* = 0.0044). The results of the present study were similar to those of previous studies [14, 15], which demonstrated that the rate and intensity of PD-L1 expression correlated with the glioma grade and were significantly higher in HGG than in LGG patients. The high PD-L1 expression in HGG patients may result in resistance to immunotherapy.

VEGF is a homodimeric protein identified as a mitogen for endothelial cells and a promoter of angiogenesis. Previous research has demonstrated that vascular proliferation correlates positively with the degree of malignancy in glioma patients [37, 38]. In agreement with these findings, we found that the immunoreactivity of VEGF increased significantly with the pathological grade. VEGF may not only promote angiogenesis and vascular permeability, but also contribute to an immunosuppressive tumor microenvironment. VEGF was reported to inhibit the maturation of dendritic cells and upregulate PD-L1 expression in tumor dendritic cells [19, 39]. In a study of 197 patients with clear cell renal cell carcinoma, PD-L1 protein expression correlated positively with VEGF expression [20]. In this study, we quantified PD-L1 and VEGF staining levels in tumor tissue exactly using Image-Pro Plus, and found a positive correlation between them. To the best of our knowledge, this is the first demonstration of a positive relationship between PD-L1 and VEGF protein expression in clinical human glioma samples. However, it is not clear whether PD-L1 and VEGF allow tumor cells to evade immune surveillance in a cooperative or independent manner by negatively regulating the immune system in glioma. Future studies in larger populations and detailed mechanistic studies of this relationship are warranted.

Monoclonal antibodies blocking the binding between PD-1 and PD-L1 have elicited significant and durable responses in several types of tumors [40]. Antibodies against either PD-1 or PD-L1 are designed to block the PD-1/PD-L1 pathway and adjust antitumor immunity to a desirable level without enhancing immunity in general [41]. Atezolizumab (a checkpoint inhibitor and antibody against PD-L1) was approved by the Food and Drug Administration for bladder cancer treatment in May 2016 [42]. The positive correlation between PD-L1 and VEGF expression has potential clinical significance for the treatment of glioma, because anti-angiogenic agents targeting VEGF or its receptors did not yield exciting results in glioma clinical trials, implying that other factors are involved in the development of glioma [43, 44]. The clinical responses to anti-VEGF treatment were transient, with tumor escape and clinical relapse usually occurring within months after an initial response. In contrast, cancer immunotherapies can have durable and striking clinical activity [45]. Thus, it is reasonable to assume that combination therapy with anti-angiogenic agents and immunotherapy would be a favorable approach. Indeed, combined therapy involving a PD-1/PD-L1 axis blockade and anti-VEGF therapy exhibited encouraging antitumor activity and tolerable adverse events in some animal models and in clinical studies [46, 47]. The combination of bevacizumab and PD-L1 inhibition (atezolizumab) appears feasible, and is being compared with sunitinib treatment in an ongoing phase III trial in previously untreated metastatic renal cell carcinoma patients (NCT02420821). MEDI4736, a human anti-PD-L1 antibody, is now being tested in combination with radiotherapy and bevacizumab for the treatment of GBM (NCT02336165).

However, it should be noted that biomarkers identifying patients who are likely to respond to PD-L1 inhibition have not yet been defined. Although PD-L1 immunohistochemistry has been approved by the Food and Drug Administration as the only predictive companion diagnostic test to determine whether pembrolizumab should be used for non-small cell lung cancer patients, improved survival outcomes have been seen in many PD-L1-negative patients [48]. In future research, the biomarker value of PD-L1 expression on tumor cells should be reassessed, and well-designed translational studies should be performed.

MMPs are a group of zinc-dependent proteins that can degrade the extracellular matrix upon their activation. One member of this family, MMP-9, is also an important promoter of tumor invasion [49]. In astrocytic glioma, the detection of MMP-9 correlated significantly with the pathological grade [50]. Recently, Colette *et al*. demonstrated that MMP-9 can proteolytically cleave PD-L1 and thus suppress T cell apoptosis [22]. In the present study, the immunohistochemical staining intensity for MMP-9 was significantly higher in HGG than in LGG. However, no significant correlation was found between PD-L1 and MMP-9 expression (*p* = 0.743). Thus, MMP-9 expression is likely to be one of several complex mechanisms that may impact the glioma immune microenvironment.

In addition, we found a strong correlation between PD-L1 expression and tumor cell proliferation, as estimated by KI-67 staining. Similarly, Ghebeh *et al*. found that the expression of PD-L1 in breast cancer patients correlated with KI-67 expression. PD-L1 expression was gradually induced in proliferating cells in parallel with KI-67, and was downregulated in quiescent cells in parallel with the absence of KI-67 [23]. However, double immunostaining with anti-PD-L1 and anti-KI-67 antibodies revealed that PD-L1 was expressed predominantly by KI-67^−^ tumor stem-like cells (lacking the KI-67 marker) derived from gliomas and medulloblastomas [14]. The expression of PD-L1 in a larger population of resting cells (KI-67^−^) may indicate that proliferation is not the only factor involved in the induction of PD-L1.

We acknowledge several limitations to our current study. First, the population enrolled in this retrospective study was relatively small, which could result in possible sample selection bias. Second, the expression of PD-L1 has been detected not only in tumor cell but also in vascular endothelial cells, which may impact on the correlation analysis of PD-L1 and VEGF. We do acknowledge the image analysis software used in their correlation analysis of PD-L1 and VEGF could not completely eliminate the interference caused by PD-L1 expressed in endothelial cells, although an AOI was drawn to exclude endothelial cells (blood vessels).

In summary, our study revealed that PD-L1, VEGF, MMP-9 and KI-67 are all overexpressed in glioma, especially in HGG. PD-L1 expression was significantly associated with the pathological grade, VEGF status and KI-67 index. As continuous variables, PD-L1, VEGF and KI-67 levels correlated positively with each other. These results indicate that PD-L1 and VEGF are involved in the cascade of the malignant progression of glioma. Thus, therapeutic regimens targeting the T-lymphocyte checkpoint and anti-angiogenesis simultaneously may yield durable responses and herald the resurgence of immunotherapy. Further investigation of the relationship between PD-L1 and VEGF will be necessary, and will contribute to the development of such therapies for human brain glioma.

## MATERIALS AND METHODS

### Ethical approval

This research was approved by the ethics committee of Shan Dong Cancer Hospital. No informed consent (written or verbal) was obtained for the retrospective analysis. Informed consent was not deemed necessary by the ethics committee, as some of the enrolled patients were deceased, and all samples were anonymous.

### Patients and specimens

We retrospectively analyzed 64 patients with glioma who underwent surgery at the Shandong Cancer Hospital Affiliated with Shandong University between June 2007 and December 2015. The glioma specimens were from 33 men and 31 women ranging from 39 to 78 years in age, with a median age of 61.3 years. All the tumor specimens were re-evaluated according to the 2016 WHO classification system by two experienced pathologists (P.L. and D.M.), with differences being resolved by careful discussion. The tumor types and grades were distributed as follows: WHO grade I pilocytic astrocytoma (*n* = 11), WHO grade II diffuse astrocytoma (*n* = 12), WHO grade III anaplastic astrocytoma (*n* = 13), and WHO grade IV GBM (*n* = 28). The inclusion criteria were as follows: a) no signs of distant metastasis; b) primary glioma confirmed through histopathology; c) well-preserved specimens; d) complete clinical records and pathologic data. Patients who had received antitumor treatment before operation, including radiotherapy and chemotherapy at the same institutions, were not included in this study. Clinical information about the patients was obtained from their medical records.

### Immunohistochemistry

Tumor specimens were surgically obtained from the 64 glioma patients. Surgically resected specimens were fixed in formalin and embedded in paraffin. The levels of PD-L1, VEGF, MMP-9 and KI-67 were evaluated by immunohistochemistry. Four-um-thick sections were deparaffinized and heated to 190°C for 5 minutes for antigen retrieval in ethylenediaminetetraacetic acid buffer (pH 9.0). After the sections were cooled, endogenous peroxidase quenching was blocked by incubation with 3% hydrogen peroxidase for 5 minutes at room temperature. Then, the slides were blocked with 5% FBS at room temperature for 15 minutes and incubated for 1 hour with primary antibodies against PD-L1 (ab 58810, Abcam, Temecula, TX, USA, 1:200 dilution), VEGF (VG1, Santa Cruz, Shanghai, China, 1:50 dilution), MMP-9 (56-2A4, Abcam, Temecula, TX, USA, 1:200 dilution) or KI-67 (MIB1, Santa Cruz, Shanghai, China, 1:50 dilution). EnVision TM+ anti-rabbit HRP labeled polymer (Dako) was used as the secondary antibody for 30 minutes of staining. Then, 3, 3′-diaminobenzidine tetrahydrochloride (DAB) was applied for color development at room temperature for 5 minutes, and sections were subsequently counterstained with hematoxylin. Parallel staining without the primary antibody was performed as a negative control.

### Evaluation of PD-L1, VEGF, MMP-9 and KI-67 staining

Two independent observers examined the stained slides in a blinded fashion. The tumor cells exhibiting PD-L1, VEGF, MMP-9 and KI-67 staining were counted under a light microscope at 200× magnification. Five random fields were examined for each tumor specimen. Staining of the cytoplasm or membranes in over 5% of tumor cells was deemed to indicate PD-L1 positivity [31]. VEGF, MMP-9 or KI-67 staining was considered positive if ≥ 10% of the tumor or endothelial cells were positive, and otherwise was considered negative [51–53].

### Quantitative analysis of IHC staining

The quantification of PD-L1, VEGF, MMP-9 and KI-67 expression levels was determined using a computerized image analysis system (Image-Pro Plus version 6.0 software). Firstly, image acquisition of IHC stained slides. Briefly, a digital image (Tag Image File Format format) with characteristic glioma on each slides was acquired under an Nikon Eclipse 80i microscopy equipped with an Nikon DS-Fi1 camera. All Images were acquired under high magnification (400×) with a constant set of imaging parameters (unified light source, exposure time, and autofocus). Secondly, optical density analysis of the images was performed with the Image-Pro Plus software. Images was calibrated and an appropriate threshold value for DAB-positive hues (brown) was set through histogram (hue; saturation; intensity). Then, an area of interest (AOI) was drawn to include most of tumor components in the image and exclude endothelial cells (blood vessels), necrosis and slice edge. After the AOI was defined, the integrated optical density (IOD) of the AOI was determined by the software. Lastly, the MOD of the selected area were calculated (IOD/area of AOI) and represented as the immunoreactivity of the candidate protein within tumor tissue. Intra-tumor heterogeneity was minimized through the elimination of the two fields with the highest and lowest MODs, and the average MOD of the remaining five fields was used to represent the PD-L1 level for an individual.

### Statistical analysis

All data were gathered in Image-Pro Plus software. Statistical analyses were performed with SPSS (version 17; SPSS, USA). The MODs from the PD-L1, VEGF, MMP-9 and KI-67 image analyses are reported as means ± standard deviations (SDs). The difference in MOD count between the two groups was assessed with a *t-test*. The correlations between PD-L1 expression and clinicopathological features were assessed with a Mann–Whitney *U* test. The correlation coefficients for the immunoreactivities of PD-L1, VEGF and KI-67 were determined with Spearman's rank correlation test. A *p* value less than 0.05 was considered significant.

## Abbreviations

GBM: glioblastoma
MOD: mean optical density
PD-1: Programmed Death-1
PD-L1: PD-Ligand 1
WHO: World Health Organization
VEGF: vascular endothelial growth factor
MMP-9: matrix metalloproteinase 9
LGG: low-grade glioma
HGG: high-grade glioma
SD: standard deviation
